# Jumbo Bacteriophages: An Overview

**DOI:** 10.3389/fmicb.2017.00403

**Published:** 2017-03-14

**Authors:** Yihui Yuan, Meiying Gao

**Affiliations:** Key Laboratory of Agricultural and Environmental Microbiology, Wuhan Institute of Virology, Chinese Academy of SciencesWuhan, PR, China

**Keywords:** jumbo bacteriophage, genome, diversity, evolution, virion structure

## Abstract

Tailed bacteriophages with genomes larger than 200 kbp are classified as Jumbo phages, and are rarely isolated by conventional methods. These phages are designated “jumbo” owing to their most notable features of a large phage virion and large genome size. However, in addition to these, jumbo phages also exhibit several novel characteristics that have not been observed for phages with smaller genomes, which differentiate jumbo phages in terms of genome organization, virion structure, progeny propagation, and evolution. In this review, we summarize available reports on jumbo phages and discuss the differences between jumbo phages and small-genome phages. We also discuss data suggesting that jumbo phages might have evolved from phages with smaller genomes by acquiring additional functional genes, and that these additional genes reduce the dependence of the jumbo phages on the host bacteria.

## Introduction

Bacteriophages are viruses that infect bacteria and are the most abundant biological entities on earth, exhibiting extremely high, uncharted diversity (Krupovic et al., 2011). Among the characterized phages, the vast majority contain genomes smaller than 200 kbp, and only 93 phages with genomes larger than 200 kbp have been isolated during the past 100 years since the discovery of phages (up to 30 June 2016). More than 80% of these were isolated during the past 3 years, which might be because of the revitalization of phage research (Reardon, 2014) and the progress in next-generation genome sequence technology in recent years. Tailed phages with genomes larger than 200 kbp are classified as “jumbo phages,” and phages of this kind usually harbor large virions. One reason for the rare isolation of jumbo phage is that the large size of the phage virions block their diffusion in semisolid medium, which prevents the formation of visible plaques (Serwer et al., 2007). The other reason is that the method used for removing bacteria with filters. Because of their large size, the jumbo phages might also be removed due to their inability to pass through the pores of the filter. Owing to their rare isolation, jumbo phages are not well known, and no systematic review on jumbo phages is currently available (Hendrix, 2009; Van Etten et al., 2010). In addition to phages with genomes larger than 200 kbp, there are also numerous phages with genomes approaching the 200 kbp size, which will not be discussed here. In this review, we summarize the characteristics, and discuss the diversity and evolution of jumbo phages.

## Distribution and hosts

Jumbo phages have been isolated from diverse environments, including water, soil, marine sediments, plant tissues, silkworms, composts, animal feces, and other unknown habitats (Table 1). Among these habitats, jumbo phages have been most frequently isolated from water environments, which might be because the liquid environments benefit the diffusion of jumbo phages and further their infection of host bacteria. Jumbo phages have most often been isolated from Gram-negative host bacterial strains (95.6%), such as strains of genera *Synechococcus* (44 phages), *Pseudomonas* (9 phages), *Caulobacter* (6 phages), *Vibrio* (6 phages), *Erwinia* (5 phages), and *Aeromonas* (5 phages). In contrast, only four jumbo phages infecting Gram-positive bacterial host strains have been isolated, and the host strains of these four phages all belong to the genus *Bacillus*. It is unclear if jumbo phage infecting only a single genus of Gram-positive bacteria is due to a special feature of *Bacillus* or just an anomaly of the small number of jumbo phages currently isolated. Further, isolation of phages infecting other Gram-positive strains and study of the interaction of *Bacillus* jumbo phage with their host strain might provide understanding for this phenomenon.

**Table 1:** **General features of jumbo phages**.

**Phage**	**Host**	**Phage taxonomy**	**Virion size (nm)**		**Genome length (nt)**	**No. of CDSs**	**No. of tRNA**	**Sample materials**	**References**
			**Head**	**Tail length**					
G	*Bacillus megaterium*	*Myoviridae*	160	453	497,513	675	20	NA^a^	Donelli et al., 1975
vs.	*Cronobacter sakazakii*	*Myoviridae*	115	118	358,663	545	26	Wastewater	Abbasifar et al., 2014
121Q	*Escherichia coli*	*Myoviridae*	116	115	348,532	611	7	Sewage	Ackermann and Nguyen, 1983
PBECO 4	*Escherichia coli*	*Myoviridae*	132	125	348,113	551	6	River water	Kim et al., 2013
K64-1	*Klebsiella pneumoniae*	*Myoviridae*	NA	NA	346,602	64	NA	NA	Pan et al., 2015
vB_KleM-RaK2	*Klebsiella sp*.	*Myoviridae*	123	128	345,809	534	5	NA	Simoliunas et al., 2013
201ϕ2-1	*Pseudomonas chlororaphis*	*Myoviridae*	129	200	316,674	461	1	Soil	Thomas et al., 2008
phiPA3	*Pseudomonas aeruginosa*	*Myoviridae*	100	185	309,208	379	5	Sewage	Monson et al., 2011
OBP	*Pseudomonas fluorescens*	*Myoviridae*	119	191	284,757	309	4	Compost	Cornelissen et al., 2012
Lu11	*Pseudomonas putida*	*Myoviridae*	124	200	280,538	391	0	Soil	Adriaenssens et al., 2012
ϕKZ	*Pseudomonas aeruginosa*	*Myoviridae*	120	NA	280,334	306	6	Sewage	Mesyanzhinov et al., 2002
CcrColossus	*Caulobacter crescentus*	*Myoviridae*	292 × 95	65	279,967	448	28	Surface water	Meczker et al., 2014
KTN4	*Pseudomonas aeruginosa*	*Myoviridae*	130	168	279,593	368	NA	Irrigated fields	KT895374
vB_EamM_Special G	*Erwinia amylovora*	*Myoviridae*	NA	NA	273,224	324	0	Branches and Blossums	KU886222.1
vB_EamM_Simmy50	*Erwinia amylovora*	*Myoviridae*	NA	NA	271,088	321	1	Bark	KU886223.1
Ea35-70	*Erwinia amylovora*	*Myoviridae*	NA	NA	271,084	318	1	Soil	Yagubi et al., 2014
PA7	*Pseudomonas aeruginosa*	*Myoviridae*	NA	NA	266,743	341	NA	Mudflat	JX233784.1
ϕR1-37	*Yersinia enterocolitica*	*Myoviridae*	138	383	262,391	367	5	Sewage	Kiljunen et al., 2005
PaBG	*Pseudomonas aeruginosa*	*Myoviridae*	136	220	258,139	308	NA	Lake water	Sykilinda et al., 2014
vB_BpuM_BpSp	*Bacillus pumilus*	*Myoviridae*	137	192	255,569	318	0	Soil	KT895374
P-SSM2	*Prochlorococcus*	*Myoviridae*	115	123	252,401	334	1	Seawater	Sullivan et al., 2005
AR9	*Bacillus subtilis*	*Myoviridae*	NA	NA	251,042	292	1	NA	Lavysh et al., 2016
ValKK3	*Vibrio alginolyticus*	*Myoviridae*	NA	NA	248,088	390	NA	Marine sediment	KP671755^b^
nt-1	*Vibrio natriegens*	*Myoviridae*	NA	NA	247,489	379	28	Marine-sediment	Comeau et al., 2014
VH7D	*Vibrio harveyi*	*Myoviridae*	NA	NA	246,964	327	NA	Seawater	NC_023568
ϕpp2	*Vibrio parahaemolyticus*	*Myoviridae*	90 × 50	110	246,421	383	30	Aquaculture waterway	Lin and Lin, 2012
KVP40	*Vibrio parahaemolyticus*	*Myoviridae*	140 × 70	NA	244,834	381	30	Marine-sediment	Miller et al., 2003
ϕEaH2	*Erwinia amylovora*	*Siphoviridae*	NA	NA	243,050	262	NA	Soil	Domotor et al., 2012
SPN3US	*Salmonella enterica*	*Myoviridae*	NA	NA	240,413	264	2	Chicken feces	Lee et al., 2011
VP4B	*Vibrio harveyi*	*Myoviridae*	NA	NA	236,053	212	NA	Ocean	KC131130.1
65	*Aeromonas salmonicida*	*Myoviridae*	NA	NA	235,229	437	16	NA	Petrov et al., 2010
vB_AbaM_ME3	*Acinetobacter baumanni*	*Myoviridae*	NA	NA	234,900	326	4	Wastewater	Buttimer et al., 2016
Aeh1	*Aeromonas hydrophila*	*Myoviridae*	NA	NA	233,234	352	27	NA	NC_005260
S-SSM7	*Synechococcus*	*Myoviridae*	NA	NA	232,878	319	5	Seawater	Sullivan et al., 2010
CC2	*Aeromonas hydrophila*	*Myoviridae*	NA	NA	231,743	427	9	Sewage	Shen et al., 2012
RSL1	*Ralstonia solanacearum*	*Myoviridae*	150	138	231,255	343	3	Soil	Yamada et al., 2010
ACG-2014f^c^	*Synechococcus*	*Myoviridae*	NA	NA	228,143	292	NA	NA	NC_026927
ϕAS5	*Aeromonas salmonicida*	*Myoviridae*	121 × 71	98	225,268	343	24	River water	Kim et al., 2012
CR5	*Cronobacter sakazakii*	*Myoviridae*	NA	NA	223,989	231	NA	NA	NC_021531
RSL2	*Ralstonia solanacearum*	*Myoviridae*	NA	NA	223,932	224	NA	NA	Bhunchoth et al., 2016
CcrRogue	*Caulobacter crescentus*	*Siphoviridae*	205 × 60	319	223,720	350	23	surface water	Meczker et al., 2014
RSF1	*Ralstonia solanacearum*	*Myoviridae*	NA	NA	222,888	230	NA	NA	Bhunchoth et al., 2016
PX29	*Aeromonas salmonicida*	*Myoviridae*	NA	NA	222,006	330	25	NA	Petrov et al., 2010
CcrKarma	*Caulobacter crescentus*	*Siphoviridae*	205 × 61	314	221,828	353	26	Surface water	Meczker et al., 2014
PAU	*Sphingomonas paucimobilis*	*Myoviridae*	NA	NA	219,372	295	7	Silkworms	NC_019521
CcrSwift	*Caulobacter crescentus*	*Siphoviridae*	219 × 63	295	219,216	343	27	surface water	Meczker et al., 2014
0305Φ8-36	*Bacillus thuringiensis*	*Myoviridae*	95	486	218,948	246	0	NA	Serwer et al., 2007
CcrMagneto	*Caulobacter crescentus*	*Siphoviridae*	211 × 58	293	218,929	347	27	Surface water	Meczker et al., 2014
ΦEaH1	*Erwinia amylovora*	*Siphoviridae*	NA	NA	218,339	241	NA	Aerial tissue	Meczker et al., 2014
ΦCbK	*Caulobacter crescentus*	*Siphoviridae*	205 × 56	300	215,710	338	26	Surface water	Meczker et al., 2014
EL	*Pseudomonas aeruginosa*	*Myoviridae*	140	200	211,215	201	NA	NA	Hertveldt et al., 2005
S-SKS1	*Synechococcus*	*Siphoviridae*	NA	NA	208,007	281	11	Seawater	NC_020851

a *NA indicated the data is not available*;

b *The GenBank accession numbers of the not published phage genomes*;

c *Forty-two isolates of phage ACG-2014f are isolated and only the information of the isolate Syn7803C90 is shown as represent*.

## Big virion and large genome size

The most notable features of jumbo phage are larger phage particles and larger genomes as compared with smaller phages. The biggest known phage is *Bacillus megaterium* phage G, which has a capsid size of 160 nm, a tail length of 453 nm, and a genome of 497 kbp in length (Table 1; Donelli et al., 1975; Kristensen et al., 2011; Drulis-Kawa et al., 2014). *B. megaterium*, the host strain of phage G, with a size of about 1.2–1.5 × 2.0–4.0 μm, can only contain ~30 virions of phage G in a single cell. As the phage's capsid size constrains the size of its genome (Hendrix, 2009), jumbo phages with big capsids can package genomes larger in size than phages with smaller capsids. Of note, the genome of phage G is only 87 kbp smaller than the genome of the smallest bacterium, *Mycoplasma genitalium* (Fraser et al., 1995).

The large genome size enables jumbo phages to contain many genes that do not exist in small-genome phages. For example, all jumbo phages have more genes responsible for genome replication and nucleotide metabolism, and some of the jumbo phages have more than one paralogous gene for DNA polymerase and RNA polymerase (RNAP; Mesyanzhinov et al., 2002; Hertveldt et al., 2005; Kiljunen et al., 2005; Thomas et al., 2007). Among the RNAPs encoded by jumbo phage genomes, most are multi-subunit RNAPs, and some of them have been found in the phage virions (Ceyssens et al., 2014; Yuan and Gao, 2016a). The structural RNAPs are mainly comprised of multiple subunits and may be injected into the host bacteria to start the immediate-early gene transcriptions before the expression of phage and host RNAPs. Transcriptomic analysis of jumbo phage infection revealed that the expression of phage genes may be dependent only on the phages' own RNAPs and independent from the host RNAPs (Ceyssens et al., 2014; Leskinen et al., 2016). Furthermore, jumbo phages also have more proteins for the lysis of the host cell-wall peptidoglycan, such as endolysin, glycoside hydrolase, and chitinase, and many of these proteins were found to be virion components with predicted functions of facilitating phage infection ability (Gill et al., 2012; Yuan and Gao, 2016a). Several jumbo phages also contain more than one tRNA gene (Table 1). For example, phage phiAS5 has 24 tRNAs that contain the anticodon sequences of 16 different amino acids (Kim et al., 2012). tRNA synthetases have been found in the genomes of several jumbo phages, such as *Yersinia* phage ΦR1-37, phage G, and so on (Kiljunen et al., 2005). The tRNAs in jumbo phage genomes are thought to correspond to codons that are abundant in phage genes, especially those encoding structural proteins, and to increase the translation efficiency of phage-specific genes (Kiljunen et al., 2005). Through their cooperative or independent action, these additional proteins encoded by jumbo phages may substitute for the function of the host proteins that are essential for the life cycle of the smaller-genome phages and reduce the dependence of jumbo phages on their bacterial hosts (O'Donnell et al., 2013). The reduction in dependence of a jumbo phage on its host bacterium might broaden the phage host range and endow jumbo phages with more chance to gain new genetic information from more bacteria by horizontal gene transfer.

## Virion composition and structure

Jumbo phages exhibit diverse virion morphology and much more complex virion structure as compared with smaller phages, including different virion sizes and specific substructures of their capsids, and tails (Fokine et al., 2005; Thomas et al., 2007). Compared with the smaller-genome phages, more structural proteins have been identified in the jumbo phages, such as 89 proteins for *Pseudomonas* phage 201Φ2-1 (four times the number of phage T4 structural proteins; Thomas et al., 2010). Another study found that *Pseudomonas* phage ΦKZ contained at least 30 phage head proteins among 62 identified structural proteins (Lecoutere et al., 2009). However, some jumbo phages only have a few structural proteins, such as 26 for *Aeromonas* phage ΦAS5 and 25 for *Ralstonia* phage ΦRSL1 (Yamada et al., 2010; Kim et al., 2012). Nevertheless, the three-dimensional structure of the jumbo phage ΦRSL1 obtained by cryo-electron microscopy showed that it had a complex head structure formed by at least five different proteins (Effantin et al., 2013).

Several jumbo phages exhibit specific virion structures. For example, the virions of phage 0305Φ8-36 and vB_BpuM_BpSp contain long, wavy, curly tail fibers, which have only been observed in a few phages (Yuan and Gao, 2016a,b). Furthermore, a spool-like protein structure called the “inner body” and encased within genomic DNA was observed in the capsid of phage ΦKZ and other jumbo phages, whereas similar structures have not been identified in smaller-genome phages (Krylov et al., 1984; Sokolova et al., 2014). The “inner body” in the phage capsid is thought to play an important role in DNA packaging and genome ejection during phage virion assembly and infection (Agirrezabala et al., 2005; Cheng et al., 2014). The large genome and virion size, the “inner body,” the wavy, curly tail fiber, and other specific structures of jumbo phages may function to facilitate phage genome packaging, the host recognition, or other processes in the jumbo phage life cycle.

## Genome organization and gene expression

The small phage genomes usually possess a modular genome structure, and genes with associated functions forming clusters (Petrov et al., 2006). However, the genes with associated functions in jumbo phage genomes are scattered or only form sub-clusters (Mesyanzhinov et al., 2002; Skurnik et al., 2012; Simoliunas et al., 2013). The timely expression of phage genes is essential for the efficient production of progeny phage. To realize the timely expression of phage genes, different phages have evolved different strategies. Similar to the small-genome phage, the genes of the jumbo phage ΦKZ are transcribed in a typical pattern, and early, middle, and late genes are transcribed in a timely manner by the phage-encoded RNAP (Ceyssens et al., 2014). By contrast, the transcriptions of phage ΦR1-37 genes does not follow the typical pattern and the majority of the genes are constitutively expressed throughout the infection process by the phage-encoded RNAPs (Leskinen et al., 2016). It is noteworthy that, for both these strategies, the regulation of phage genes is under the control of phage-encoded RNAPs, but not the host RNAPs.

## Classification and evolution

**Figure 1:**
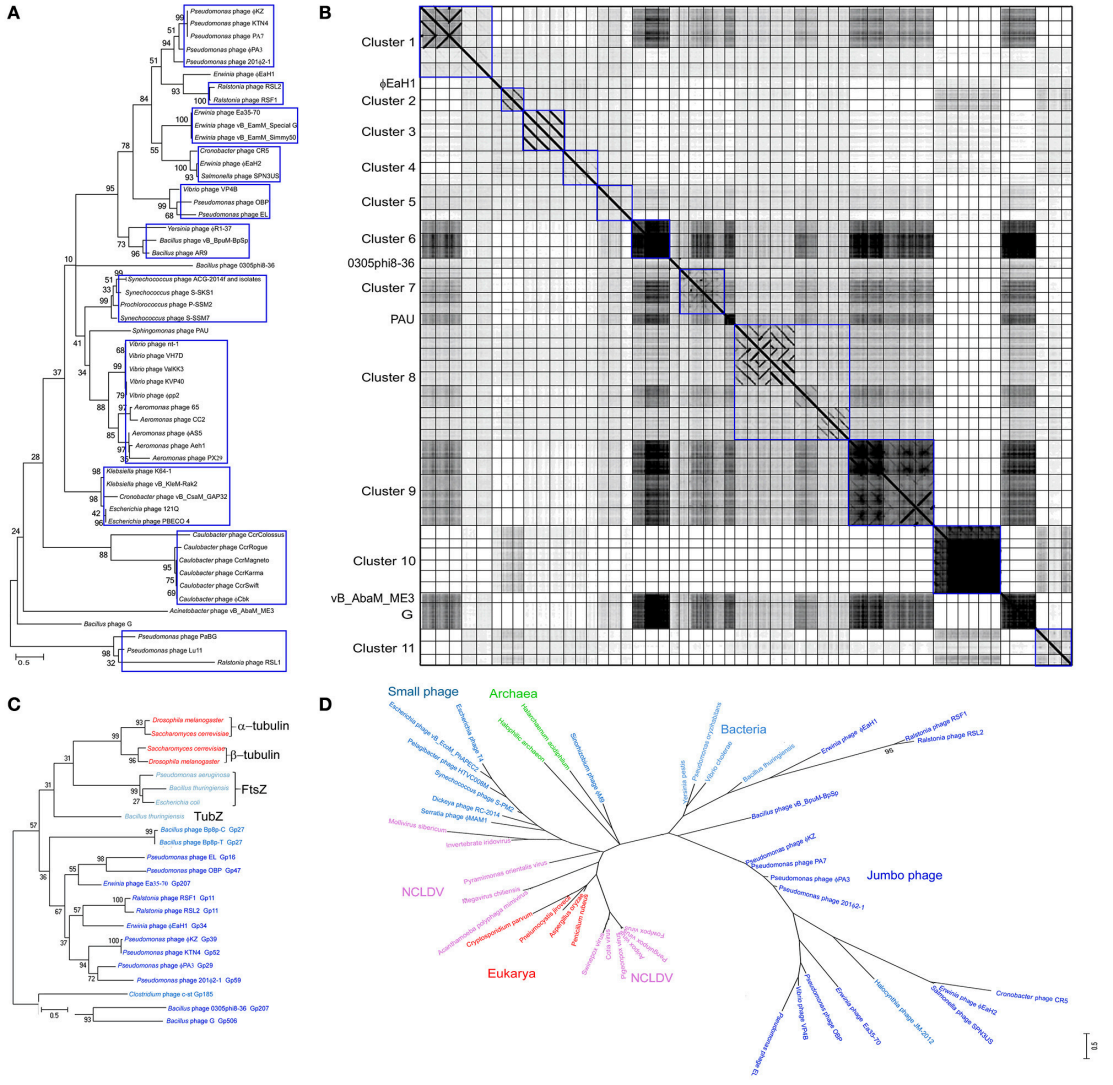
**Phylogenic and comparative genomic analysis of Jumbo phages**. The amino acid sequences of the terminase large subunit from 93 jumbo phages **(A)**, the tubulin-like protein from Jumbo phage, bacteria, fungi, and phage with genome near 200 kbp **(C)**, and the B-family DNA polymerase from jumbo phage, small phage, bacteria, archaea, eukarya, and NCLDVs **(D)**, were used for phylogenetic analysis, respectively. The amino acid sequence were alignment by Muscle and the tree were constructed by Maximum Likelihood method with a bootstrap of 1,000 using Mega 6.0 (Tamura et al., 2013). **(B)** The genome of 52 Jumbo phage were compared by using Gepard (Krumsiek et al., 2007). The phage genome are arrangement in the same order as in Figure 1A. Phages belonging to different clusters are showed in rectangle boxes.

The evolution of jumbo phages has not been well characterized owing to their rare isolation, unavailability of sufficient jumbo phage genomes, and the high genome divergence. To date, based on the morphology similarity and the host range, only some jumbo phages were classified as ΦKZ-like phages (Krylov et al., 2007) and T4-like phages (Petrov et al., 2010), respectively, while no solid genetic evidence is available for the classification of these jumbo phages. Lots of jumbo phages have been designated as a new lineage based on their low genome homology with previously characterized phages (Hardies et al., 2007; Krylov et al., 2007; Yamada et al., 2010; Adriaenssens et al., 2012; Simoliunas et al., 2012; Meczker et al., 2014). Phylogenetic analysis based on the amino acid sequences of the terminase large subunits from 93 jumbo phages revealed that the jumbo phages could be classified into 11 clusters and five singletons (Figure 1A). Comparative genomic analysis of the jumbo phages by using Gepard (Krumsiek et al., 2007), which calculates the similarity of genome sequences and show the similar DNA fragments (word length of 10 and window size of 0) as dot plots, also showed that the jumbo phage could be classified into the same 11 clusters and five singletons (Figure 1B). Based on the phylogenetic and comparative genomic analysis, some phages that used to be classified as ΦKZ-like phages, such as phage Lu11, phage OBP, and phage EL, are now classified into different clusters in this study. Core gene analysis of the jumbo phage also showed that the phage which used to be classified in T4-like phage group should be classified into new cluster. For example, although phage ΦPAS5 has been classified in the T4-like phage group, it only shares 26% core genes with T4 phages (Kim et al., 2012). Otherwise, phage ΦPAS5 and Aeh1, which are classified into the same cluster in this study, share 90% of their genes (Kim et al., 2012). The jumbo phages from each cluster usually infect host strains from the same species or the same genus, and some phages of the same cluster have been isolated from similar ecological environment.

Although the jumbo phages from each cluster showed relatively high genomic similarity (higher than 15%), the phages from different clusters exhibited extremely low or no similarity, suggesting that the jumbo phages have divergent origins. According to previous reports, jumbo phages might be derived from the smaller-genome phages by acquiring novel genetic information and further increasing their genome size and genome function over evolutionary time (Hendrix, 2009). Analysis of the core genes between jumbo phages and small genome phages revealed that the genes essential for phage life cycle are existing both in jumbo- and small-phage (Miller et al., 2003; Kim et al., 2012). Genomic analysis of phage 0305Φ8-36 revealed that the phage genome might be fused from two ancestral virus genomes via the horizontal exchange of a genome module (block of genes) during the evolutionary process (Hardies et al., 2007), while the majority of the jumbo phages might obtain genes from their host by horizontal gene transfer to form larger genomes (Burkal'tseva et al., 2002).

Apart from the jumbo phages, whose propagation mechanism is mainly unclear, there are other large dsDNA viruses include poxviruses, asfarviruses, iridoviruses, ascoviruses, and phycodnaviruses, defined as nucleocytoplasmic large dsDNA viruses (Iyer et al., 2006), and giant viruses that infect amoeba, including mimiviruses, marseilleviruses, pandoraviruses, pithoviruses, faustoviruses, and *Mollivirus sibericum* (Forterre and Gaia, 2016). The replicative cycle of these large and giant dsDNA viruses include the presence in the host cytoplasm of viral factories that produce the progeny viruses (Netherton and Wileman, 2011). Such viral factories were hypothesized to be at the origin of the modern eukaryotic nucleus (Forterre and Gaia, 2016). Jumbo phages exhibit similar replication characteristics to the eukaryotic NCLDVs. The tubulin-like protein PhuZ of phage 201Φ2-1 can form a spindle and position the phage genome DNA to the mid-cell region of the bacterial host; subsequently, the encapsidated DNA forms a rosette-like structure surrounded by a larger DNA mass, which, to some extent, resembles the viral factory of NCLDVs (Kraemer et al., 2012). Proteins homologous to PhuZ have also been found in the genomes of several jumbo phages and phages with genomes near 200 kbp. Phylogenetic analysis of the homologous proteins of PhuZ reveals that the jumbo phages are evolutionary closely to phages with genome near 200 kbp, but distinct from the small genome phages and the cellular microorganisms (Figure 1C). The evolutionary relationships of jumbo phage based PhuZ-like protein are consistent with that based on the terminase large subunit (Figure 1A) and the B-family DNA-polymerase (Figure 1D). Though the smaller-genome phage do not encode tubulin-like protein in their own genomes, they also engage the tubulin-like protein from the host bacteria to facilitate the phage genome replication (Munoz-Espin et al., 2009). Formation of viral factory-like structures by jumbo phages and large viruses creates a platform to concentrate virus replication-associated proteins, virus genomes, and host proteins required for replication, and also protects viruses from host defenses (Netherton and Wileman, 2011), which might benefit the virus propagation. Except for the feature of forming viral factories, NCLDVs and giant viruses of amoeba also have more genes associated with genome replication, nucleotide metabolism, and some other biochemical processes (Legendre et al., 2014). Although jumbo phages, NCLDVs, and giant viruses of amoeba exhibit several similar features, they are evolutionary distant (Figure 1D). The jumbo phages are much more closely related to the bacteria and archaea, while the NCLDVs show a closer evolutionary relationship with the eukaryotes.

## Conclusion and perspective

More recently, larger viruses have been isolated, and their discovery has greatly enriched our understanding of biological entity diversity and evolution (Bhunchoth et al., 2015; Sharma et al., 2015). Jumbo phages have been isolated from diverse niches and exhibit extremely high genetic diversity. However, generally speaking, the jumbo phages exhibit several common features that differentiate them from the smaller-genome phages. First, the jumbo phages have notably bigger virions and larger genomes. Second, the genomes of the jumbo phages form non-modular structures, and genes with associated functions are scattered throughout the genome. Third, they contain more genes associated with biochemical processes and more than one paralog of essential genes for the phage life cycle. Fourth, they contain structural RNAPs in phage virion with the function of controlling jumbo phage gene expression. Fifth, the jumbo phages are evolutionarily distant from the small genome phages. Despite the common features that differentiate them from smaller-genome phages, jumbo phages show more divergent characteristics among each other, such as low genome similarity, individual virion substructure, and different propagation mechanisms.

For the purpose of archiving a greater understanding of the jumbo phages, several areas need to be studied further. First, isolation and complete genomic sequencing of more jumbo phages. In order to isolate novel jumbo phages, re-isolation of environmental samples by reducing the agar concentration in the upper medium or a deep metagenomic sequencing of environmental samples may be effective. Second, further study the interaction mechanism between jumbo phages and their host bacteria, including the phage propagation mechanism. Our current knowledge of phages is mainly based on the study of smaller-genome phage. Although the jumbo phages might have evolved from the smaller-genome phages, they show many differences from the smaller-genome phages in terms of genome structure and propagation strategy. Third, functional analysis of the genes with more than one paralog and the structural RNAPs. The additional paralogous genes and structural RNAPs might reduce the dependence of jumbo phages on their host bacteria. However, the functions of these genes for the jumbo phage life cycle have mainly been ascribed based on the bioinformatic analysis, and no experimental evidence is available. Functional analysis of these genes will provide a greater understanding of the phage-host interaction and evolution of jumbo phages. Fourth, analyze the evolution and the origin of jumbo phages. The large genomes of jumbo phages are thought to have evolved from small phage genomes by acquisition of novel genetic information during the evolutionary process, which led to a reduced dependence of phages on their host strain. Study of the evolution and origin of jumbo phages could provide knowledge for understanding the origin of cellular biological entities and the evolution of biological entities from cell-dependent to cell-independent status.

## Author contributions

YH designed and drafted the manuscript. YH and MG revised the manuscript. All author approved of the final content of the manuscript.

## Funding

This study was supported by the National Natural Science Foundation of China (No. 31500155, 31170123).

### Conflict of interest statement

The authors declare that the research was conducted in the absence of any commercial or financial relationships that could be construed as a potential conflict of interest.
